# Design and Optimization of ECG Modeling for Generating Different Cardiac Dysrhythmias

**DOI:** 10.3390/s21051638

**Published:** 2021-02-26

**Authors:** Md. Abdul Awal, Sheikh Shanawaz Mostafa, Mohiuddin Ahmad, Mohammad Ashik Alahe, Mohd Abdur Rashid, Abbas Z. Kouzani, M. A. Parvez Mahmud

**Affiliations:** 1 Electronics and Communication Engineering Discipline, Khulna University, Khulna 9208, Bangladesh; ashik.ece140903@gmail.com; 2 ITI/Larsys/Madeira Interactive Technologies Institute, 9020-105 Funchal, Portugal; sheikh.mostafa@tecnico.ulisboa.pt; 3 Department of Electrical and Electronic Engineering, Khulna University of Engineering and Technology, Khulna 9208, Bangladesh; ahmad@eee.kuet.ac.bd; 4 Department of Electrical and Electronic Engineering, Noakhali Science and Technology University, Noakhali 3814, Bangladesh; marashid.eee@nstu.edu.bd; 5 School of Engineering, Deakin University, Geelong, VIC 3216, Australia; abbas.kouzani@deakin.edu.au (A.Z.K.); m.a.mahmud@deakin.edu.au (M.A.P.M.)

**Keywords:** ECG modeling, ECG generator, Gaussian function, optimization, data compression

## Abstract

The electrocardiogram (ECG) has significant clinical importance for analyzing most cardiovascular diseases. ECGs beat morphologies, beat durations, and amplitudes vary from subject to subject and diseases to diseases. Therefore, ECG morphology-based modeling has long-standing research interests. This work aims to develop a simplified ECG model based on a minimum number of parameters that could correctly represent ECG morphology in different cardiac dysrhythmias. A simple mathematical model based on the sum of two Gaussian functions is proposed. However, fitting more than one Gaussian function in a deterministic way has accuracy and localization problems. To solve these fitting problems, two hybrid optimization methods have been developed to select the optimal ECG model parameters. The first method is the combination of an approximation and global search technique (ApproxiGlo), and the second method is the combination of an approximation and multi-start search technique (ApproxiMul). The proposed model and optimization methods have been applied to real ECGs in different cardiac dysrhythmias, and the effectiveness of the model performance was measured in time, frequency, and the time-frequency domain. The model fit different types of ECG beats representing different cardiac dysrhythmias with high correlation coefficients (>0.98). Compared to the nonlinear fitting method, ApproxiGlo and ApproxiMul are 3.32 and 7.88 times better in terms of root mean square error (RMSE), respectively. Regarding optimization, the ApproxiMul performs better than the ApproxiGlo method in many metrics. Different uses of this model are possible, such as a syntactic ECG generator using a graphical user interface has been developed and tested. In addition, the model can be used as a lossy compression with a variable compression rate. A compression ratio of 20:1 can be achieved with 1 kHz sampling frequency and 75 beats per minute. These optimization methods can be used in different engineering fields where the sum of Gaussians is used.

## 1. Introduction

Cardiovascular disease (CVD) is one of the leading causes of morbidity and mortality around the globe. About 80% of CVD deaths take place globally in low- and middle-income countries. About 92.1 million U.S. adults are currently suffering from CVDs. A total of 17.3 million people died globally in 2013 due to CVDs [1]. By 2030, the projected percentage of having some form of CVDs is 43.9%. Therefore, to save millions of people, CVD diagnosis and prediction are significantly important. The electrocardiogram (ECG) is a low cost and non-invasive test and has the ability to detect various CVDs such as myocardial infarction (heart attack) and arrhythmias. This is why ECG is commonly used as diagnostics and prognostic tools for CVDs. Modeling different cardiac dysrhythmias through ECGs could help to understand the cardiac conditions. It can also help to create better features that could be used to distinguish cardiac dysrhythmias from the machine learning perspective. Motivated by these, this paper presents simplified mathematical modeling for generating different patterns for cardiac dysrhythmias.

Different modeling techniques have been developed over the decades for the modeling of ECGs. The homomorphic analysis and modeling of typical ECG signals have been conducted by the pole-zero model [2]. Another pole-zero model utilized the poles and zeros to form clusters, and the clusters can be related to the constituent waves of the ECG [3]. Another study optimized the rational functions and their poles for ECG signal using particle swarm optimization [4]. The pole-zero system’s problem is that it cannot find a satisfactory solution because it involves finding an optimum point on a (potentially) multimodal error surface [5]. The Chip Away Decomposition (ChAD) algorithm, an iterative method for Gaussian parameter determination, is used to decompose and represent the ECG model. However, the model does not perform well in the presence of noise [6]. Besides, it is assumed that there is no meaningful baseline wander within a cardiac cycle, which is not always valid in a real situation, and it has to start from a baseline point [6]. A modified version of the Van der Pole (VdP) equation has been used for natural cardiac pacemaker by utilizing a modified version of the FitzHugh–Nagumo model [7,8]. A dynamical 3-D state-space canonical model involving heart rate variability and low-frequency oscillations associated with Mayer waves has been proposed by McSharry et al. [9].

Other modeling techniques, such as the Fractional-order modeling technique, have been used to develop a generalized ECG signal generation template. Variations of ECG waveforms under normal and abnormal heart conditions have been described by two different model classes using coupled VdP oscillators [10]. The ECG codebook model (ECGCM) has been applied to extract useful features for automatic detection of Myocardial Infarction. ECGCM reduces ECG dimension and contains more meaningful semantic data for Myocardial Infarction detection [11]. The Generalized Orthogonal Forward Regression (GOFR) technique was applied for automatic ECG wave extraction and morphology tracking. GOFR with a specific function (Gaussian Mesa function (GMF) or Bi-Gaussian function (BGF)) provides an informative model to track the morphology of the waves [12]. ECG signals were reconstructed using exponentially damped sinusoids (EDS) parameter and the linear prediction coefficients using the inverse transform [13]. A dynamical model was used to process and segment the ECG signal into its components (P, Q, R, S, T). A signal decomposition model-based Bayesian filtering method has been introduced by Roonizi et al. [14]. ECG components have been utilized as hidden state variables and estimated simultaneously as a time series through an extended Kalman smoother (EKS) [14]. A genetic Fuzzy classifier system model increases ECG classification accuracy regarding more precise arrhythmia detection [15]. A new segmented method for modeling the ECG signal with Hermitian basis functions yielded half in compression compared to the non-segmented method [16]. Using only the QRS complex of Hermite features for pattern recognition [17,18], and compression [19,20] have also been proposed [16]. Other modeling techniques such as polynomial approximation [21] and parametric modeling using discrete cosine transform [22] have also been developed for modeling and compression. 

The above models are complex to produce useful ECG signals. However, in the discussed literature, ECG components using Gaussian could be a promising solution. Encouraged by this, in this paper, a simplified mathematical model with optimization is developed for producing various kinds of ECG rhythms. The major contributions and key topics covered by this research are as follow:

- A simplified mathematical model based on the sum of two Gaussians has been proposed, which does not require baseline adjustment [23], and it is straightforward compared to the current models.
- Most fitting or optimizing techniques are designed to calculate only one Gaussian function, which is not ideal for the proposed method. Therefore, to solve these problems along with the model, two-hybrid optimization methods have been proposed.
- The proposed model’s performance is evaluated using time domain, frequency domain, and time-frequency domain analysis.
- Among various applications, one of the most promising ones is the ECG generator for education and research purpose. A graphical user interface (GUI) has been developed to show the proposed system’s potential use. Besides, noisy ECG generation and data compression have also been presented.

This paper is organized as follows. Firstly, this study’s background problem is formulated with the proposed ECG model through hybrid optimization methods in Section 2. In Section 3, data collection and processing for the performance evaluation of the test model and hybrid optimization are discussed. The results and comparison with real data are presented in Section 4. In Section 5, two applications have been discussed. The discussion and conclusion are presented in Section 6 and Section 7, respectively.

## 2. Background Problems and Proposed Model

**Figure 1:**
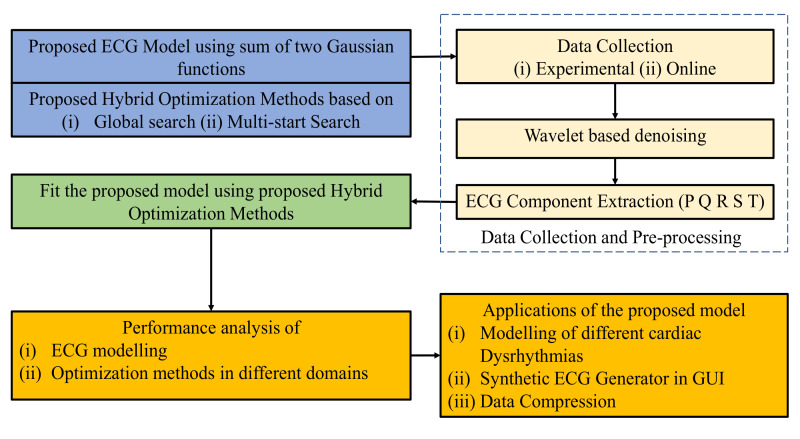
Proposed flow diagram of the study.

The overall flow diagram of the study is shown in Figure 1. First, the proposed ECG model and hybrid optimization methods are presented with their background problems. After that, the proposed model is fitted with real ECG using hybrid optimization methods. Finally, the performance measurements and applications of the proposed model are discussed in details.

An ideal ECG is a combination of P, Q, R, S, and T waves; thus, the proposed simplified mathematical model is generated by constructing and assembling these waves. ECG modeling has two problems: (1) designing a simplified mathematical model with a minimum number of model parameters representing a full ECG signal, and (2) finding optimal model parameters representing different cardiac dysrhythmias. The proposed model along with the limitation of the current model (Section 2.1) and the proposed hybrid optimization technique (Section 2.2), are discussed below.

### 2.1. Proposed Model

ECG components, i.e., *P, Q, R, S*, and *T* waves, have an approximately symmetric “bell curve” shape that quickly falls toward both sites. This ‘’bell curve” shape is one of the reasons for the widely used Gaussian wave [24]. Moreover, a small number of parameters can be represented by a Gaussian function and has also been used in other biomedical signal modeling, e.g., photoplethysmogram (PPG) [25]. Awal et al. [24] use non-uniform number Gaussian functions to represent ECG components. One Gaussian function is used for P wave, while the sum of two Gaussians is used to describe *Q* wave. This non-uniformity of the number of Gaussians in the model creates an extra classification problem where different ECG signal components need to be classified before modeling.

A uniform equation for all ECG components $i\in \left(P,Q,R,S,T\right)$ is proposed, and the equation of generating ECG wave can be written as:

(1) $$ECG_{model}=\overset{j=2}{\underset{i\in P,Q,R,S,T;\;j=1}{\sum }}A_{i,j}\;e^{-\left[\frac{{\left(t-t_{i,j}\right)}^{2}}{\sigma_{i,j}^{2}\;}\right]\;}\;+\;c_{i}$$

where $A_{i}$ is the height of the curve’s peak, $t_{i}$ controls center position of the peak, $\sigma_{i}$ controls the width of the ECG, and j=1, 2 represents the number of Gaussians. Optimal $A_{i}$, $t_{i}\;$ and $\;\sigma_{i}$ need to be selected, which is an optimization problem, and it is discussed in the next section. Note that $c_{i}$ is an additional parameter that can be used to control baseline and noise modeling. 

### 2.2. Optimization Problem Formulation and Proposed Optimization Method

The performance of the mathematical model is vastly dependent on the choice of parameters. Generally, it is done by fitting the mathematical model to real-world ECG signals. Given a uniformly spaced discrete-time data points $\left\{t=1,\;2,\ldots ,N\right\}$ associated with ECG∈P,Q,R,S,T data values, where N is the total number of discrete-time points or samples in each ECG beat, and the sum of two Gaussians stated in Equation (1) is needed to fit the real ECG data to minimize the root mean square error (RMSE). Mathematically, it can be written as:

(2) $$RMSE=\sqrt{\frac{1}{N}\overset{N}{\underset{t=1}{\sum }}{\left(ECG_{Model}\left(t\right)-ECG_{real}\left(t\right)\;\right)}^{2}}<\varepsilon$$

where $ECG_{Model}\left(t\right)$ is the proposed model having seven controlling parameters: $\left\{A_{1},t_{1},\sigma_{1},A_{2},t_{2},\sigma_{2,\;}C_{i}\right\}$ for each ECG component, i.e., P,Q,R,S,T. $ECG_{Model}\left(t\right)$ needs to fit $ECG_{real}\left(t\right)\;$ so that minimizes RMSE given in Equation (2). The proposed model is the sum of two Gaussians. Goshtasby and O’Neill showed that Gaussian parameters ($A_{i},\;t_{i},\;\sigma_{i}$) can quickly and accurately be determined when a model or function contains only one Gaussian from its zero-crossings [26] or in a deterministic way, for example, using Crauna’s, Guo’s, and Roonizi’s methods [27,28,29]. However, it is difficult to accurately determine the position and standard deviation of all Gaussians from its zero-crossing when a function contains two or more Gaussians [26]. Our proposed model consists of the sum of two Gaussians; therefore, a better optimization method is required to tune parameters.

**Figure 2:**
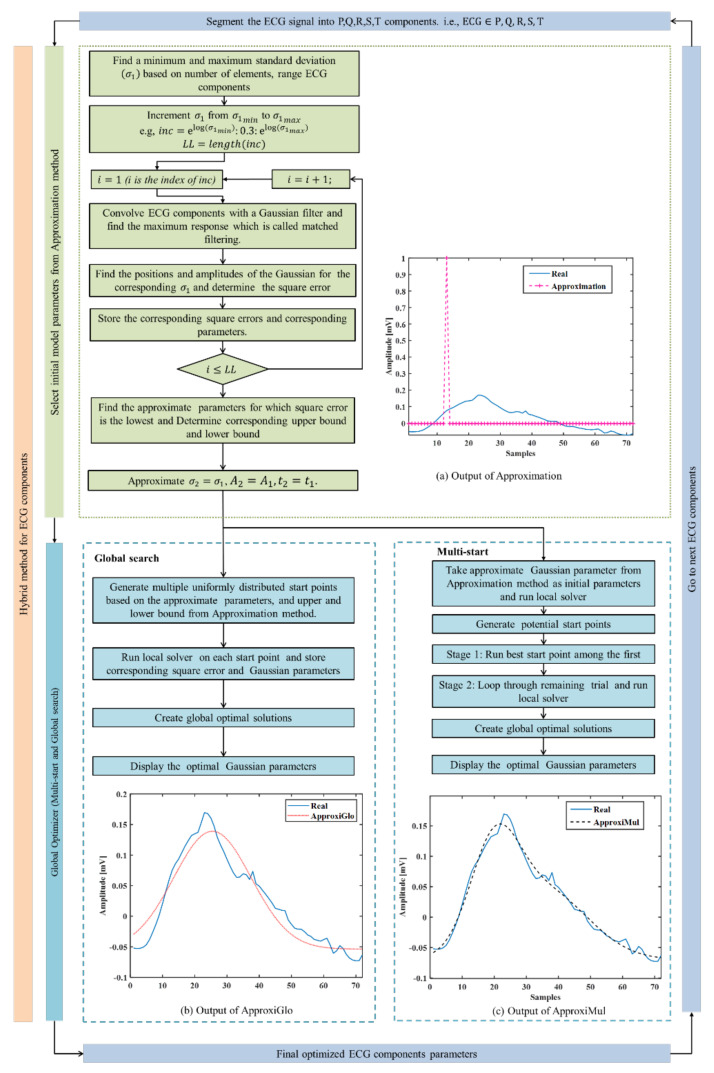
Proposed hybrid optimization technique. (**a**) Step 1: Approximation of the Gaussian parameters. In this step, the effect of adding multiple Gaussians is not taken into account. Step 2: These estimated parameters are regarded as initial parameters in the (**b**) Multi-start and (**c**) Global search optimization solver to determine the optimal Gaussian parameters.

Two hybrid optimization methods (ApproxiGlo and ApproxiMul) have been proposed. Both methods are comprised of two steps (see Figure 2): (1) Determination of initial parameters and corresponding lower and upper bounds, i.e., approximation method; (2) Determination of final optimal Gaussian parameters by a global optimization solver from the approximate parameters and corresponding limits calculated in step 1. In this paper, two global optimization solvers are explored: (**a**) Multi-start and (**b**) Global search. In summary, ApproxiGlo comprises the Approximation method and Global search, whereas ApproxiMul comprises the Approximation method and Multi-start; see Figure 2.

Step 1: An approximation method for selecting initial model parameters is used. These initial parameters are used as input parameters for the global optimization solvers in Step 2. A flow diagram for the approximation method is shown in Figure 2a, and a brief description is given below:

⮚ Step 1.1 After Segmenting ECG, beat into P, Q, R, S, T components. i.e., ECG∈P,Q,R,S,T, minimum and maximum values of $\sigma_{1}\;$ are calculated based on the number of samples. Assume the total time duration of an ECG component $x_{R}$ and the number of samples in this time duration is $N_{s}$, the $\sigma_{1_{min}}$ and $\sigma_{1_{max}}\;$ can be approximated by the following equations:
  (3) σ1min=xRNs5σ1max=xR3
⮚ Step 1.2 Increment the values of $\sigma_{1}$ from $\;\sigma_{1_{min}}$ to $\sigma_{1_{max}}$ at an interval of 0.3 and run through all the different values. Then, construct a Gaussian filter with the Gaussian as follows:
  (4) $$G_{filt}=e^{-\left[\frac{S^{2}}{\sigma_{1}\left(i\right)}\right]}\;$$
  where S is a vector calculated from
  (5) $$S=-\left(\lceil \frac{N_{s}}{2}\rceil +1\right):1:⎣\left\lceil \frac{N_{s}}{2}\right\rceil ⎦$$
  here, ⌈.⌉ denotes the ceiling function used to convert the floating number into an integer. This arrangement helps to integer increment and reduce the number of iterations.

Convolve ECG with the Gaussian filter and find the maximum response using the matched filtering approach. After that, find the positions $t_{1}$ and amplitudes $\;A_{1}$ of the Gaussians for the corresponding $\sigma_{1}\;$ and determine the square error.

⮚ Step 1.3 Store the corresponding RMSE and corresponding parameters.
⮚ Step 1.4 Repeat step 1.2 and step 1.3 for all values of $\sigma_{1}$.
⮚ Step 1.5 Select the parameters ($A_{1},\;\sigma_{1},\;t_{1}$) for which RMSE is the lowest.
⮚ Step 1.6 Finally, replicate the parameter of the second Gaussian using the calculated Gaussian parameters, i.e., $A_{2}=A_{1},\;\sigma_{2}=\sigma_{1},\;t_{2}=t_{1}$.

As the second Gaussian parameter is approximated from the first Gaussian, this step is considered the Approximation method. 

Step 2: To increase the accuracy of the Approximation method, a global optimization solver is chosen. Among various global optimization solvers such as multi-start, global search, genetic algorithm, simulated annealing, particle swarm, etc., multi-start and global search are simple, fast, and easy to use. Hence, these are adapted to obtain the optimal Gaussian parameters. Multi-start and global search have a nearly similar approach to finding the multiple-minima or global minima, and both algorithms start a local solver from multiple start points. The multi-start optimizer uses uniformly distributed start points within bounds or user-supplied start points, whereas the global search uses a scatter-search mechanism for generating start points.

A real ECG P wave is fitted by the model parameters calculated from this approximation method and represented in Figure 2. From Equation (3), it can be seen that the parameters $\sigma_{1}$ and $\sigma_{2}$ are approximated, hence called the Approximation method. Figure 2 (output of (a)) illustrates the model fitting using the approximate techniques, and Figure 2 (output of c) and (output of d)) represents the model fitting results of the proposed hybrid optimization techniques on a real ECG component, P wave.

## 3. Databases and Pre-Processing

Denoising is required as the real data is prone to noise, and data formatting is necessary to fit the model. The collected data are pre-processed, and then ECG components are extracted for modeling. A brief description is given below about these data collection and pre-processing.

### 3.1. Data Collection

Real ECG was used for fitting the model and testing it for accuracy. To ensure the diversity of data sources and provide the model with different cardiac dysrhythmias, ECG data was collected from (i) Biomedical Signal Processing Lab, Khulna University of Engineering and Technology (KUET), Khulna, Bangladesh, and (ii) two publicly available online databases. The first one was collected from a volunteer, and others were obtained from accessible online databases. Combining these three ECG data collection methods ensures the diversity of data sources and different cardiac dysrhythmias.

#### 3.1.1. Experimental Data Collection

Experimental ECG data was collected from a 26-year-old volunteer with no known cardiovascular disorder (normal subject). The volunteer was informed about the experiment and asked to relax to guarantee lower motion artifact and EMG signal on the data. BIOPAC data acquisition unit (MP36) [30] with BIOPAC electrode lead set (SS2L) and disposable vinyl electrodes (EL503) were used for data collection setting; details can be found in [31].

#### 3.1.2. Data Collection from Online

The collected data from KUET was a completely healthy subject, i.e., ECG was normal. Therefore, it is not possible to model different cardiac dysthymias from only a normal ECG. Thus, ECG from MIT-BIH Arrhythmia Database was also used, recorded by the Beth Israel Hospital Arrhythmia Laboratory between 1975 and 1979, and 60% of the total data are from patients who exhibited different cardiovascular diseases. This database contains both normal and abnormal beats, and both of them were used to justify how well the proposed methods work on different cardiac dysrhythmias and commonly used for ECG modeling and classification with their original sampling frequency [32]. For this work, 100 series from MIT-BIH Arrhythmia Database were used. These have 23 datasets with 360 Hz sampling frequency, and each of them is slightly over 30 minutes. These files are also annotated for different cardiac dysrhythmias with age, sex, and medication [33]. A publicly available database, University College Dublin Sleep Apnea (UCDSA) database, is also added. This polysomnographic data contains ECG and other physiological signals such as electroencephalogram (EEG), Electrooculogram (EOG), etc. We used only ECGs with a sampling frequency of 128 Hz from ten subjects (Record Number: ucddb002, ucddb003, ucddb005, ucddb007, ucddb009, ucddb010, ucddb011, ucddb013, ucddb014, ucddb015). Note that the sampling rate of each dataset was kept to its original sampling frequency. We have not done any up-sampling and down-sampling. Our intention is to show how well our proposed model and optimization methods work under different sampling frequencies. This indicates that the model is independent of sampling frequency and generalization ability to replicate different cardiac dysrhythmias under diverse sampling frequency.

### 3.2. Denoising

It is inevitable that the wanted signal is prone to mixing with various noises such as white noise, pink noise, baseline wander, muscle noise and motion artifact, and other noises, which in varying degrees cause misjudgment and omission of conventional ECG identification. In this study, the noise was reduced by the Discrete Wavelet Transform (DWT) based filtering. The Coiflet mother wavelet [34] of order 6 with 8 levels of decomposition using adaptive shrinkage rule [31], together with a single level rescaling and soft thresholding strategy, was used for denoising.

### 3.3. ECG Components Extraction

**Figure 3:**
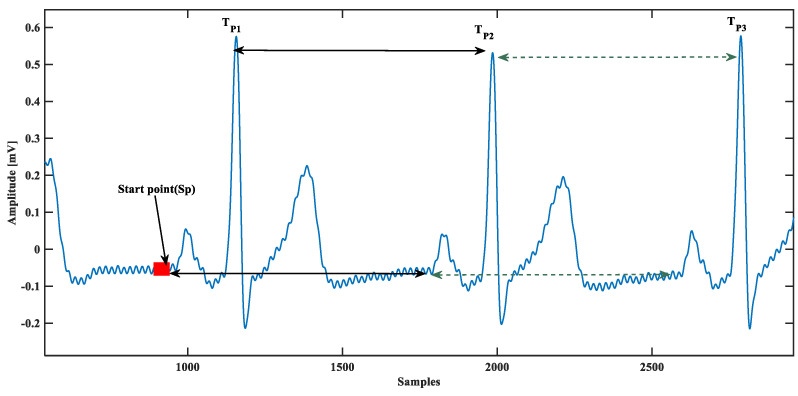
Automated electrocardiogram (ECG) beat separation from the database.

The proposed model is based on a single ECG beat, so the single beat ECG isolation is essential; this was done using beat time calculated from QRS complex peaks. First, visually a starting point $\left(Sp\right)$ of the database was chosen, assuming that it was the beginning of that particular ECG beat because the database could start in the middle of an ECG beat. Next, the peak of the QRS complex was located $\left(Tp_{1},\;Tp_{2},\ldots \ldots Tp_{N}\right)$ and the ECG beat was isolated by:

(6) $$ECG_{Beat}=ECG_{Database}\left[u\left(t-Tp_{N-1}+S_{p}\right)-u\left(t-Tp_{N}+S_{p}\right)\right]$$

where $u\left(t\right)$ is a unit step function, and t  is the time. This concept is almost similar to [28], except they use it for P-wave detection. Instead of having any starting point defined visually, they started with zero crossings, as shown in Figure 3. Using the zero-crossing technique, the automatic starting point is prone to misdetection of P-wave accurate starting for several reasons such as baseline wander and power-line interference, and other noises. A practical scenario presented in Figure 3 reveals that there could be several false zero-crossing before starting the P-wave. Therefore, due to accuracy concerns, a visual stating point was chosen instead of the automatic starting point.

## 4. Results

The optimization performance of ApproxiGlo and ApproxiMul is presented first. Then, the results of different cardiac dysrhythmias modeling are evaluated and compared with contemporary state-of-the-art methods.

**Figure 4:**
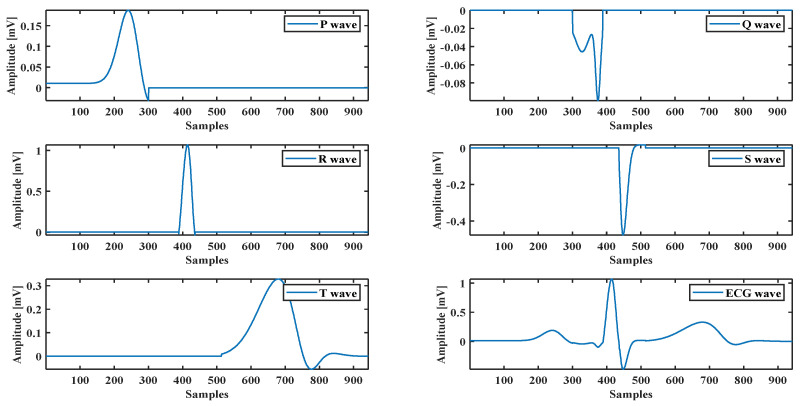
ECG components and a complete ECG signal.

A healthy subject’s ECG recorded by the BIOPAC data acquisition system is used to evaluate the optimization method’s performance and the proposed model. It also uses an ECG waveform selected from the MIT-BIH database [35]. The model parameters are calculated by fitting the model using hybrid methods. For example, the ECG P wave presented in Figure 2 shows how the proposed model with the optimization method fits with the real ECG P wave. The same technique is used for all ECG components (see Figure 4).

**Figure 5:**
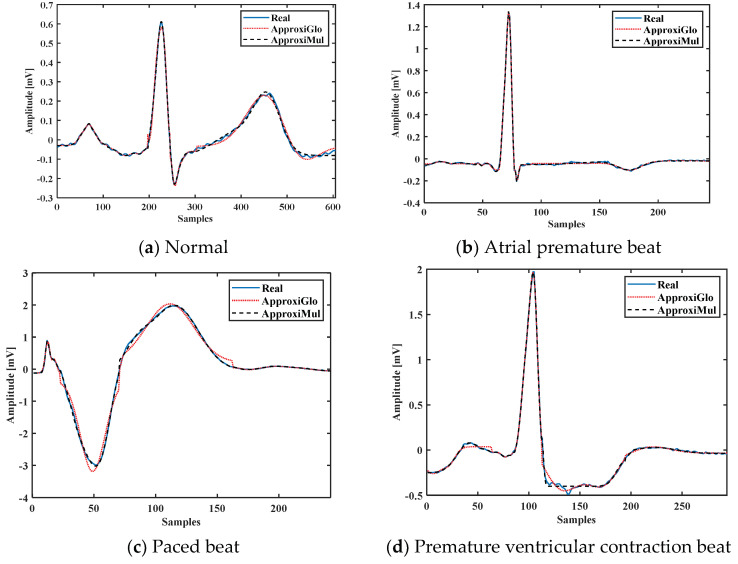
Model fitting of different cardiac dysrhythmias which are morphologically different and have different pathophysiological conditions.

**Figure 6:**
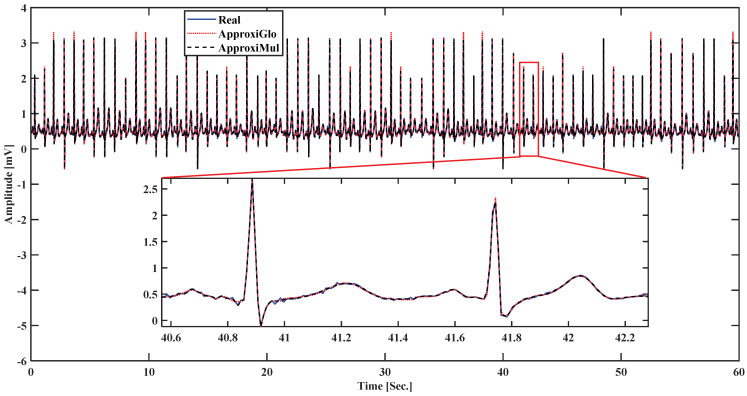
Model fitting on a 1-min ECG collected from UCDSA.

To demonstrate the proposed model’s efficiency, a wide variety of ECG beat is used, such as normal, atrial premature beat, paced beat, and premature ventricular contraction ECG beat collected from MIT-BIH database; see Figure 5. It is found that the proposed model can fit adequately not only the normal ECG beat but also can fit different cardiac dysrhythmias. Besides, a 1-min ECG collected from UCDSA is presented in Figure 6 to show the performance of our proposed method in a long and multi-beat situation. The inner Figure 6 shows the zoomed version.

### 4.1. Performance in Time Domain

The proposed model is evaluated by visual inspection and assessed in the time domain, frequency domain, and time-frequency domain. The proposed two-hybrid optimization methods tune the model parameters, and ECG beats are finally created by using optimized model parameters to evaluate the average model performance in different domains. The relationship between the model parameters and the physiologically and morphologically different ECG waveforms are evaluated using the goodness of fitting in time-domain by using Mean Square Error (MSE), Normalized MSE (NMSE), Root MSE (RMSE), and Normalized Root MSE (NRMSE). The required equation of MSE, NMSE, RMSE, NRMSE, and Correlation coefficient (CORR) has been given in Appendix A.1. The minimum value of MSE, NMSE, RMSE, and CORR’s maximum value indicates that the model can mimic the real-world different ECG waveform with physiological accuracy. Table 1 represents the average goodness of fitting value when the model fits Normal ECGs collected from MIT-BIH Arrhythmia database and UCDSA database using ApproxiGlo and ApproxiMul. It also compares with the nonlinear fitting method presented in [24]. Both ApproxiGlo and ApproxiMul provide improved results compared to the nonlinear fitting method.

Besides, normal ECG beats, collected from MIT-BIH Arrhythmia database and UCDSA database, atrial premature beat, paced beat, and premature ventricular contraction beats are also used to fit the proposed model. It can be seen that the optimization using ApproxiMul has a lower error compared to ApproxiGlo (Table 2 and Table 3) Though the difference between ApproxiGlo and ApproxiMul is not significant, in the case of Premature Ventricular Contraction ApproxiGlo has fewer errors. Both methods have a high correlation coefficient of greater than 0.9.

### 4.2. Performance in Frequency Domain

**Figure 7:**
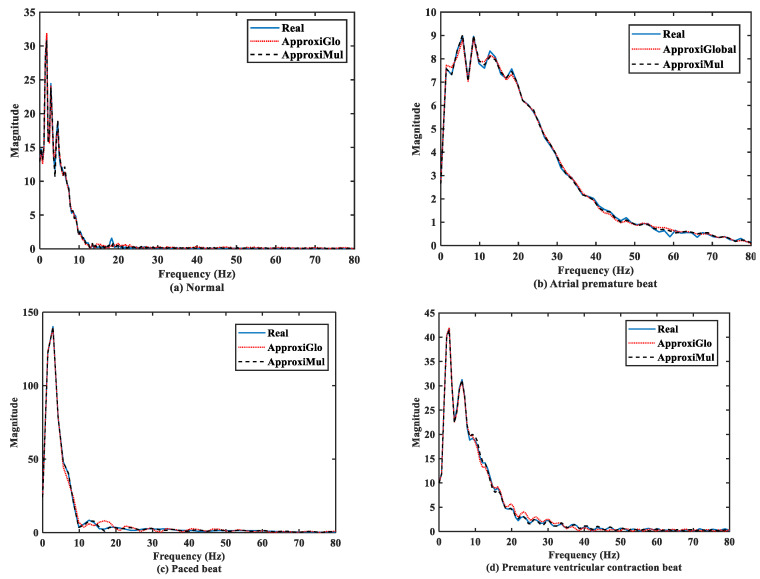
FFT of different types of ECG beats representing physiological or pathophysiological conditions.

**Figure 8:**
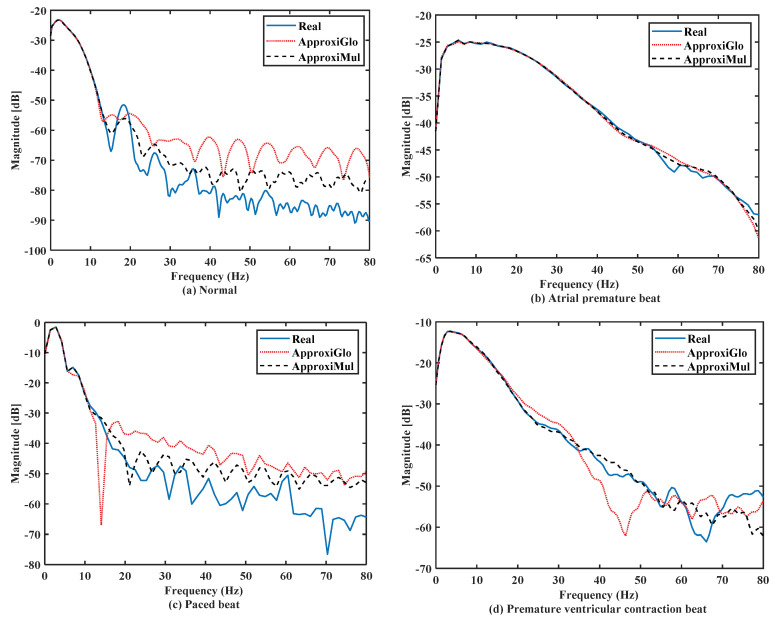
PSD of different types of ECG beats representing physiological or pathophysiological conditions.

The significant frequency component of ECG lies between 0 Hz and 50 Hz [6,36]. It is found that both the model and real ECG signals offer almost the same frequency response in the frequency domain (Figure 7). It can be seen that the normal ECG has a higher amplitude with a lower dominant frequency band of 0 Hz to 10 Hz, which is opposite to the atrial premature beat where the dominant frequency band lies between 0Hz to 80Hz. On the other hand, paced beat shows a higher amplitude with a lower dominant frequency content. The premature ventricular contraction beat shows a moderate amplitude with the dominant frequency between 0 Hz and 30 Hz. In contrast, other frequency-domain measurements, such as PSD, can provide more information. Figure 8 shows that below 50 Hz normal and atrial premature beats have an almost same frequency response in both methods and real ECGs. However, in the case of ApproxiGlo, one severe downward peak occurred at the 14.06 Hz and 46.41 Hz in the PSD plot for pace beat and premature ventricular contraction beat, which is unwanted. This phenomenon does not happen with ApproxiMul, which indicates better synchronization than ApproxiGlo. It enables the ECG model to stay within its allocated spectrum or band of frequency and avoid interfering with other frequency components. If proper timing is not maintained, the amplitude and width of *P, Q, R, S,* and T in ECG will be changed and lead to misdiagnosis. Moreover, generally different cardiac dysrhythmias like tachycardia and bradycardia are nothing but the various periods or frequency which must be maintained to ensure the perfect modeling. As the model can match the modeled ECG frequency with the real ECG, whatever its frequency components, it means a better model for both normal and abnormal ECG.

### 4.3. Performance in Time-Frequency Domain

**Figure 9:**
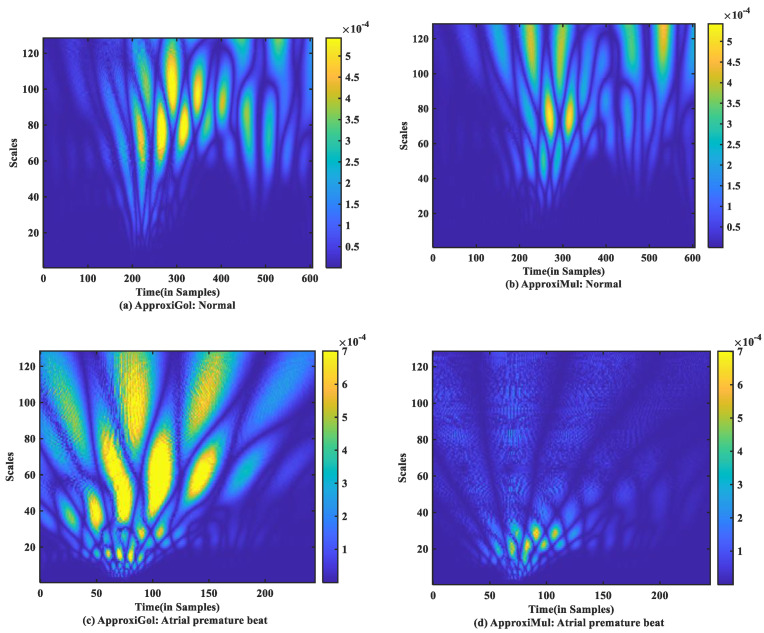

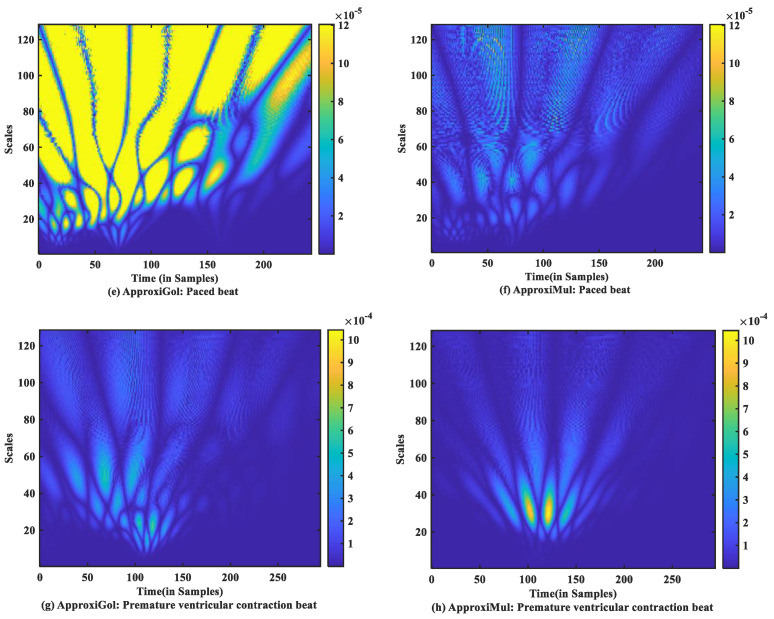
Absolute scalogram difference of different types of ECG beats representing physiological or pathophysiological conditions using ApproxiGlo and ApproxiMul methods.

For time-frequency measurement, scalogram difference (ScD) is chosen to see the time-varying spectral difference between real and model ECG. To illustrate, in the case of normal ECG modeling using ApproxiGlo (Figure 9a), the *Q*, *R*, *S* exhibit higher ScD in the range between $2.5\times 10^{-4}\;$ and $5\times 10^{-4}$ over a more extended scale interval of 60 to 120 scales. This value of ScD is lower in the case of P and T waves. This happens due to the fact that the QRS complex has a higher amplitude and frequency contents than *P* and *T* waves [37]. The ScD is lower using ApproxiMul (Figure 9b) than the ApproxiGlo method in normal ECG modeling. In the same way, atrial premature beat and other different physiological or pathophysiological conditions can be interpreted. Overall, ApproxiGlo has more energy difference in the time-frequency domain than the ApproxiMul except for the premature ventricular contraction. The worst occurred in the paced beat when it was compared with the ApproxiMul. However, in the case of premature ventricular contraction shown in Figure 9d, the ScD’s energy is higher using ApproxiMul than ApproxiGlo, which is also expected from the system’s time and frequency analysis.

## 5. Applications of the Proposed Model

There are several applications for the proposed model. The two most obvious applications are: (i) the ECG generator for simulation purposes and (ii) ECG compression. 

### 5.1. Synthetic ECG Generator

**Figure 10:**
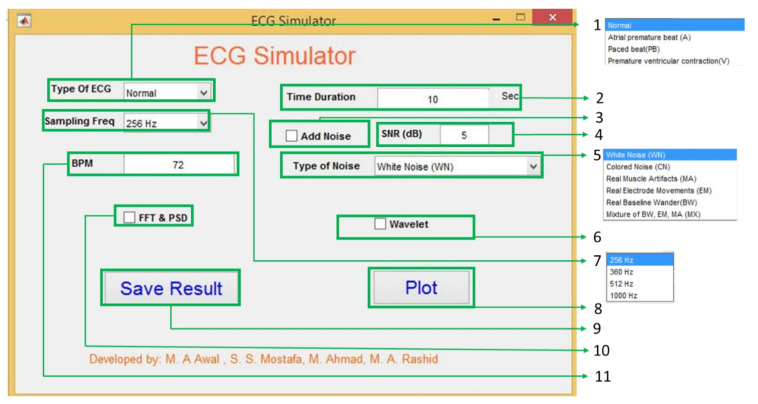
Graphical user interface for the ECG generator.

A graphical user interface (GUI) in Matlab has been developed to show the proposed system’s potential use (Figure 10). Using the sum of two Gaussians model parameters, ECG signals are created with user desired requirements such as different types of ECG signals with different sampling frequency, noise type and strength, and user-defined beat rate (BPM).

(1) Type of ECG: The GUI has four different ECG types: normal, atrial premature beat, paced beat, and premature ventricular contraction. The ECG coefficients are calculated from the collected data using BIOPAC [30] and the MIT-BIH database [33]. The coefficients’ value for one single ECG beat is shown in the appendix used to generate ECG.

(2) Time Duration: If the user wants to generate the ECG with a fixed time, it is possible using this text box. It accepts positive integer values in seconds. By default, the system always generates ECG for 10 seconds.

(3) Add noise: Checking and unchecking enables and disables the noise adding options. Only allowing this option to Signal-to-Noise Ratio (SNR, 4 in Figure 10) and types of noise (5 in Figure 10) is useful in the system. Without enabling this option, the user’s values and options for the SNR and types of noise do not affect the generated ECG.

(4) SNR (dB): To give users more flexibility, the SNR of the generated ECG can be controlled by putting values in the text box. The GUI considers the value in dB and calculates the noise power using Equation (7).

(7) $$Noise\;Power=\frac{P_{y}}{10^{\frac{SNR}{10}}}$$

here, $P_{y}$ denotes the signal power. For a N-point ECG signal $y\left(n\right)$, the signal power is measued as the energy per sample, and mathematically it can be expressed as $P_{y}=\frac{1}{N}\overset{N-1}{\underset{n=0}{\sum }}{\left|y\left(n\right)\right|}^{2}$.

(5) Type of noise: As noise cancellation in ECG is a popular research topic among researchers. The noise adding option is added to the GUI. ECG generation is the reversed process of the calculation of the model parameter so $c_{i}\;$ in Equation (1) can be used as a noise parameter, $d\left(t\right)$.

(8) $$ECG_{Noisy}=\overset{j=2}{\underset{i\in P,Q,R,S,T;\;j=1}{\sum }}\;A_{i,j}\;e^{-\left[\frac{{\left(t-t_{i,j}\right)}^{2}}{\sigma_{i,j}^{2}\;}\right]\;}\;+d\left(t\right)$$

**Figure 11:**
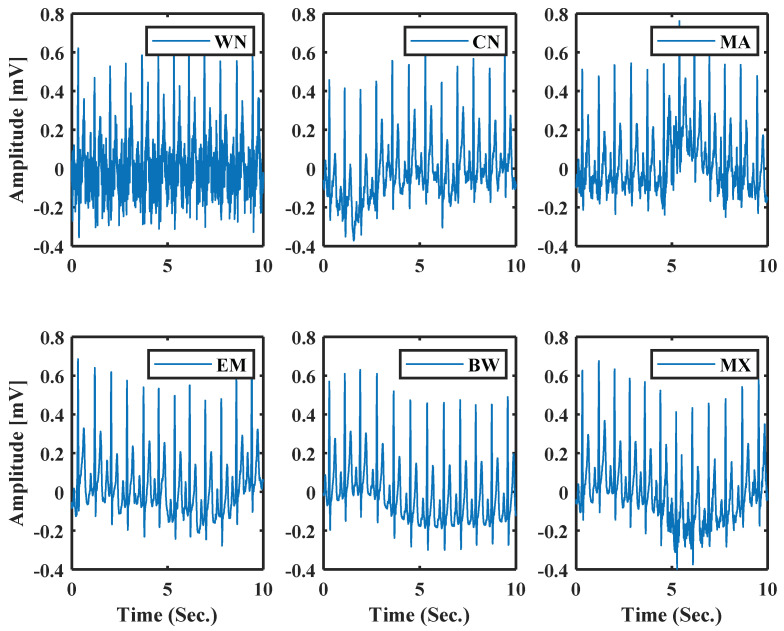
Normal ECG with different types of noise using the model parameters.

Using this parameter as a noise parameter, the model can support both synthetic noise (simulated) and real noise. This noise parameter can help to generate a more realistic ECG with different noise types of noise. Six types of noise are chosen to show the effectiveness of this approach. They are White Noise (WN), and Colored Noise (CN) generated from a mathematical model, and Real Muscle Artifacts (MA), Real Electrode Movements (EM), Real Baseline Wander (BW), Mixture of BW, EM, MA (MX) are from MIT-BIH noise stress test database [38]. Figure 11 shows normal ECG generated using model parameters with different noises; the system uses the same process as [31].

**Figure 12:**
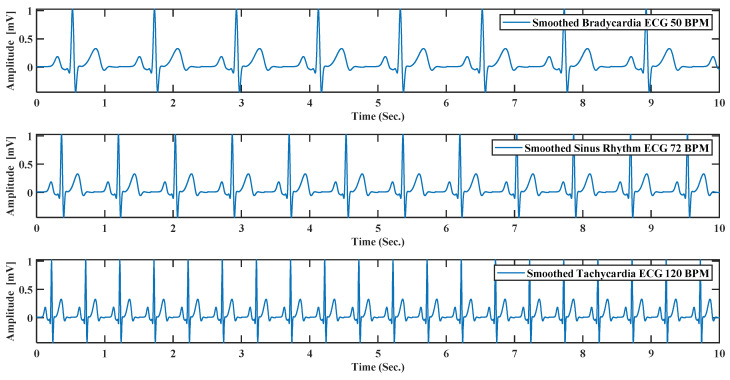
ECG simulation by model for 10 s. For bradycardia with BPM (beat per minute) 50, sinus rhythm with BPM 72, tachycardia with BPM 120.

(6) Wavelet Analysis: The wavelet analysis is also be performed by using this GUI.

(7) Sampling frequency: There are four different sampling frequencies which the user in this GUI can choose are 256 Hz, 360 Hz, 512 Hz, and 1000 Hz. Though the model’s sources are fixed frequency (BIOPAC is 1 kHz and MIT-BIH is 360 Hz), the frequency variation is done by resampling, which applies an antialiasing FIR lowpass filter in the desired frequency. By doing so, it is understandable that the model is independent of source sampling frequency.

(8) Plot: The plot button is for showing the generated ECGs. 

(9) Save Result: Save result is also a button for saving the generated ECG in mat format so that later the user can use it as s/he wants to use it.

(10) FFT & PSD: This checkbox enables and disables the FFT and Power spectral density (PSD). By seeing these two, the user can understand the frequency domain property of the model generated ECG.

(11) BPM: This text box can do beat per minute or BPM of the generated ECG by default; the system always generates 72 BPM ECG signal. As bradycardia and tachycardia are nothing but less or high BPM by changing the beat rate, it is possible to create bradycardia and tachycardia like Figure 12.

### 5.2. ECG Compression

The ECG data is converted into model parameters by modeling the ECG, and later, the ECG beat can be recreated from these model parameters. Therefore, this transform can be treated as lossy compressing [39]. On the other hand, it can remove the noise without any extra work. The compression ratio (CR) is a ratio of both signals’ length. For discussion, a single minute ECG signal is used because, in ECG, bpm (*beats per minute*) is usually used to represent heartbeat. If a subject has HR bpm and the signal has $N_{sample}$ sample per second (Hz) then the CR ratio can be written as:

(9) $$CR=\frac{Original\;Size}{Size\;After\;Modelling}=\frac{N_{sample}\times 60\times B_{p}}{\lceil HR\rceil \times \left(N_{G}\times N_{S}+N_{SS}\right)\times B_{p}}=\frac{N_{sample}\times 60}{\lceil HR\rceil \times N_{S}\times \left(N_{G}+1\right)}$$

In the case of the original one-minute ECG, $N_{sample}$ is multiplied by 60, which gives numbers needed to represent the one-minute signal. Each of these numbers representing ECG samples is multiplied with $\;B_{p}$ which denotes the precision bits size. On the other hand, modeled ECG signal representation does not depend on sample numbers; instead, it relies on the number of model parameters and heart rate. For successfully recovering ECG beat proposed model needs seven Gaussian parameters $\left(N_{G}=7\right)$ for each segment (P, Q, R, S, T) multiplied by the number of segments $\left(N_{S}=5\right)$ plus a number of size segment $\left(N_{SS}=\;N_{S}\right)$. The result of that should be multiplied by the number of beats (HR) and $B_{p}$. As the model parameters and sample ECG both are float point, in this case, the same precision bits $\left(B_{p}\right)$ is assumed. 

For the compression, in a practical scenario, the ECG beat could be a fraction (70.80), and the segment, in that case, the number of segments, is to round to the next segment of the last ECG beat. The worst-case scenario has to round to next beat ⌈HR⌉. Here, instead of regular HR worst-case scenario heart rate ⌈HR⌉ is used. It is considered that sampling frequency is known; both have an equal header in the file. Different studies presented in Table 4 show that different ECG sampling frequencies are shown to produce a correct diagnosis. Mahdiani et al. showed a 50 Hz sampling rate is enough for visual inspection and calculating time-domain heart rate variability parameters with R-peak deformity [40]. A similar finding of 250 samples per second, causing no significant differences with the reduction in peak amplitudes, is also found [41]. On the other hand, Abboud et al. [23] showed for spectral analysis high sampling rate (1 kHz) is necessary, and in this work, the collected normal ECG is at 1 kHz. The range of heart rate variability can be from bradycardia (for example, HR = 50), and tachycardia (for example, HR = 120), then CR ranges from 30 to 12.5. A similar type of compression done by a different model has more than 7:1 compression ratio in 128 Hz sampling frequency [39]. Clifford et al. [39] have a better compression ratio than the proposed methods. However, the dynamic model is much more complex as well as symmetric asymmetric turning points are needed to be identified. The typical BPM is considered 75 and compares the proposed model with other methods in Table 4. 

## 6. Discussions

In this paper, a simplified model for generating different patterns for cardiac dysrhythmias is proposed. Two hybrid optimization methods also optimize model parameters. The model can produce different beats such as normal, atrial premature beat, paced beat, and premature ventricular contraction. It is logical to discuss the proposed model’s salient features and the limitation and future works of the current model. One of the salient features is the ability to model asymmetric ECG components. For example, the ECG T-wave is asymmetric, and the P wave is slightly asymmetric. The proposed model can replicate such asymmetricity as the model uses the sum of two Gaussians. Due to symmetricity, a single Gaussian is not able to reproduce asymmetricity. Moreover, abnormal ECG P-wave such as P mitrale (*P mitrale is a sign of left atrial enlargement, usually due to mitral stenosis*), P Pulmonale (*P Pulmonale is a sign of right atrial enlargement, usually due to pulmonary hypertension (e.g., chronic respiratory disease)*), multifocal atrial rhythms cannot be produced by a single Gaussian wave but can be produced by our model. 

Most of the methods are complex, and results are shown qualitatively by graphical presentations [9,39,46]. A comparison is presented in Table 5 with additional information and the unique characteristics of each study. Suppaplola et al. used  M number of Gaussian where M is determined by zero-crossing and based on NRMSE [6]. Hence, the model is stochastic in nature and increases the model parameters as M  is not fixed [6]. The same issues arise in Parvaneh’s study [47]. The number of Gaussian was extended up to 133 for better accuracy [47]. It has no baseline wonder parameters.

On the other hand, Clifford et al. used the 3D state-space model and with baseline wander parameters. They have used 5 to 7 Gaussians to model the whole ECG signal [9,39,46,48]. Clifford et al. [39] used 6 Gaussian functions to model one beat ECG signal using a Nonlinear least-squares solver. On the other hand Clifford et al. [46] n+2m number of Gaussian functions where, n = Symmetric turning point, m = asymmetric turning point. They represented each ECG component with single Gaussians except for T wave. Due to asymmetric turning point, two Gaussians have been employed to model only ECG T wave. However, not only T wave, other ECG components such as P wave is slightly asymmetric [49]. Roonizi and Ebadollah [28] develop a faster non-iterative method to fit Gaussian Function Riding on the Polynomial Background. Dubois et al. [12] used mainly Gaussian Messa Function (GMF); however, for T-wave bi-Gaussian function (BGF) function was used for better performance. Badilini et al. [50] use 6 Gaussian Messa Function (GMF) to represent ECG. The fitting was done by Generalized Orthogonal Forward Regression (GOFR). GMF has more parameters than the single Gaussian function, where a single Gaussian function has three settings GMF has five.

The same type of work is also done by Dubois et al. [51]. Some other works, such as Elda et al. [32] use polynomial functions to model ECG rather than Gaussian function. However, the number of Gaussian used by the proposed model is higher than some published work. In comparison with the literature, the proposed model can solve ECG components’ asymmetric problem using the sum of two Gaussians. It is also uniform for all ECG components, so there is no need to separately classify or identify different ECG components. The developed hybrid optimization performs better than the most used nonlinear methods. A summary is presented in Table 5. Besides, we compare our two optimization methods with other state-of-the-art Gaussian fitting methods such as Crauna’s method [27], Guo’s method [52], and the Fast, Accurate, and Separable (FAS) method [29]. Different performance metrics are calculated, and results are shown in Table 6. Note that we have added run-time as a measure of computational load. We have run the program 100 times on normal ECG to accomplish the task, and the average results are presented. The run-time is calculated on a core i9 processor having 64GB RAM. It can be seen from Table 6 that our proposed ApproxiMul provided the best performance in all metrics except in the run time. The FAS method showed the lowest run-time but delivered the worst performance in MSE, NMSE, RMSE and, CORR. Therefore, giving more importance to accuracy and precision than run-time, our proposed ApproxiMul method provided the highest results. By looking closer the ApproxiMul process, it can be seen that ApproxiMul comprises the Approximation method and multi-start method. The approximation method is faster and more time is consumed in the multi-start optimization as it is a global optimization algorithm used to find out the global minimum point.

Our proposed method is simple, easy to implement, and has localization capability, meaning that it can simulate both long-duration ECG signal on a beat-by-beat basis and even ECG component by component basis. This feature can be helpful to detect and diagnose some diseases such as sleep apnea. However, there are some limitations to this study. The flaws of the proposed recommendations are discussed below:

- In this study, ECG∈P,Q,R,S,T components are extracted manually. However, this problem can be solved by the method presented in [53,54,55].
- Another limitation of this study is the number of ECG beats used. Few ECG beats were taken into account for model fitting and optimization. However, this work aims to find out an optimization method for ECG model fitting and the possible use of this optimized model in simulating different cardiac dysrhythmias, for example, atrial premature beat, paced beat, etc.
- The automatic classification of different types of ECG beats can be possible to implement by our proposed method. In that case, model parameters can be treated as features. These features can be trained by fuzzy-hybrid neural networks [56], support vector machine (SVM) [57,58], light gradient boosting machine [59], and Bayes maximum-likelihood (ML) classifier [32]. In addition to that, prominent features can also be selected by some feature selection algorithms such as the mRMR method and the Jaya algorithm [60,61,62] to increase the classification accuracy.
- A dictionary can be built based on our model and represent and classify cardiac dysthymias (which is called matching pursuit) [63].
- The proposed model can be used in model-based signal denoising [39].

## 7. Conclusions

This paper proposes a simplified mathematical model for generating an ECG signal in different cardiac dysrhythmias. In addition to that, two hybrid methods are proposed and compared with the non-linear fitting and other optimization algorithms. The experimental results show that the proposed model can replicate the essential features of human ECG. The model and optimization methods are tested on three different datasets having different sampling frequencies and show outperforming results in every dataset. This indicates that the model is independent of sampling frequency and has the generalization ability to replicate different cardiac dysrhythmias. The model fits the normal ECG with an average MSE of 0.0023 and atrial fibrillation with an average MSE of 0.0291, which indicates the effectiveness of this simplified model. Moreover, many morphological changes, such as atrial premature beat, paced beat, and premature ventricular contraction, can be fitted by selecting proper model parameters. With the baseline drift factor in the model, this model can fit the real ECG effectively. A Matlab-based- GUI is developed to show the potential use of the proposed model. This model can also achieve a data compression ratio as high as 20:1 in 1 kHz sampling frequency and outperformed other studies in high sampling rate. A small number of ECG beat types were taken into account for model fitting and optimization. This work aims to find out an optimization method for ECG model fitting and the possibility of using this optimized model to simulate various cardiac dysrhythmias other than the fitted cardiac dysrhythmias, for example, ventricular hypertrophy, ventricular fusion beat, etc. The proposed model can be used as a supplementary medical education tool, testing, and simulating intracardiac signals. In this work, the physiological problem related to changes in beat-by-beat overtime is not discussed. Some diagnostic problems, e.g., sleep apnea, can be visible in long-duration ECG, such as in 1 minute or 5-minute duration by detecting bradycardia and tachycardia events. However, the model can replicate such long-duration phenomena by simulating each ECG beat and fit each time-varying ECG beats over time. Besides, the long-duration ECG comprised of the normal and abnormal beat can also be simulated by the model using beat by beat basis. Even the changes in ECG components could be simulated due to the localized nature of the model. 

Interestingly, the optimization of Gaussians can not only be used in ECG signals but can also widely be used to represent many natural phenomena and industrial processes. For example, Gaussians can model an approximation of the airy disk in image processing, microscopic applications, fluorescence dispersion in flow cytometric DNA histograms, and laser heat source in laser transmission welding. Therefore, the proposed model and optimization method can also be used in those applications. 

## Figures and Tables

**Table 1:** Comparison between the proposed ApproxiGlo and ApproxiMul and the nonlinear fitting method described in [24] for normal ECGs.

Average Goodness of Fitting	Nonlinear Fitting Method (MIT-BIH Arrhythmia Database) [24]	Proposed Hybrid Method (MIT-BIH Arrhythmia Database)		Proposed Hybrid Method (UCDSA Database)	
		ApproxiGlo	ApproxiMul	ApproxiGlo	ApproxiMul
MSE	0.00779	0.00071	0.0001	0.0007	0.0003
NMSE	0.172477	0.01724	0.0031	6.4025 × 10^−6^	2.1465 × 10^−6^
RMSE	0.0882615	0.02657	0.0112	0.0267	0.0164
NRMSE	0.029748	0.1313	0.0555	0.0089	0.0054
CORR	0.9205	0.99016	0.9983	0.9969	0.9988

**Table 2:** Comparison between ApproxiGlo and ApproxiMul for normal and atrial premature beat.

Normal(N)			Atrial Premature Beat (A)		
Average goodness of fitting	ApproxiGlo	ApproxiMul	Average goodness of fitting	ApproxiGlo	ApproxiMul
MSE	0.00071	0.0001	MSE	6.29 × 10^−5^	3.33 × 10^−5^
NMSE	0.01724	0.0031	NMSE	0.00175483	0.00092763
RMSE	0.02657	0.0112	RMSE	0.00793334	0.005768
NRMSE	0.1313	0.0555	NRMSE	0.04189072	0.03045697
CORR	0.99016	0.9983	CORR	0.9991193	0.99953455

**Table 3:** Comparison between ApproxiGlo and ApproxiMul for paced beat and premature ventricular contraction beat.

Paced Beat (PB)			Premature Ventricular Contraction (V)		
Average goodness of fitting	ApproxiGlo	ApproxiMul	Average goodness of fitting	ApproxiGlo	ApproxiMul
MSE	0.0141746	0.0011756	MSE	0.00028384	0.00075337
NMSE	0.0096177	0.0007977	NMSE	0.00173033	0.00459269
RMSE	0.1190572	0.0342873	RMSE	0.01684748	0.02744755
NRMSE	0.0980699	0.0282432	NRMSE	0.04159728	0.0677694
CORR	0.9951467	0.9995984	CORR	0.99912836	0.9976848

**Table 4:** Comparison of ECG compression methods.

Method	Frequency (Hz)	BPM	CR
Turning Point (TP) [42]	200	75	2:1
Peak-Picking (spline) with Entropy coding [43]	500	75	10:1
DPCM-Linear Prediction Interpolation and Entropy Coding [44]	500	75	7.8:1
DWT using variable length code [45]	360	75	22.19:1
DWT using direct binary representation [45]	360	75	23:1
Polynomial transform (PT) [21]	500	75	12.6 (N = 100)
A dynamical model [39]	128	60	>7:1
Proposed method	1 k	75	20:1
	200	75	4:1
	500	75	10:1
	360	75	7.2:1
	128	60	3.2:1
	128	75	2.56:1

**Table 5:** Comparison of different ECG methods using a Gaussian function.

**Reference/Studies**	Number of Gaussians	Additional Information	Type of Algorithm to Fit the Model	Outcomes
Suppappola et al. [6]	Between 6 and 14 (for normal ECG)	Chip away decomposition (CHAD) algorithm. Variable number of Gaussians. Prone to noise.	Three optimization methods used: Nelder Mead simplex method, Newton-Raphson and steepest descent method.	NMSE ≤ 10%
Clifford et al. [39]	6	A 3-D model. Complicated model. Asymmetry of T-wave is not considered. Can be stuck in local minima due to the use of local optimizer i.e. *lsqnonlin*.	Nonlinear least-squares solver using *lsqnonlin* function.	Presented visually
McSharry et al. [9]	5	3-D model. Complicated model. Asymmetry of T-wave is not considered.	Experimental search	Presented visually
Clifford [46]	Adaptive determination for p = n+2m (n = Symmetric turning point, m = asymmetric turning point)	A dynamical model. Asymmetry of T-wave is considered. Can be stuck in local minima due to the use of local optimizer, i.e., *lsqnonlin*.	Nonlinear least-squares solver using *lsqnonlin* function	Presented visually
Parvaneh and Pashna [47]	Manually or Automatically (up to 133)	Gaussian function. Variable number of Gaussians. Complexity increases as the number of Gaussians increases.	Zero-crossing and minimum bank method	The best area under absolute of local error = 233.2
Badilini et al. [50]	6	Gaussian Messa Function (GMF) (consists of 5 parameters). GMF has more parameters than the single Gaussian function.	Generalized orthogonal forward regression (GOFR)	(CORR = 0.96) QT
Dubois et al. [51]	6	GMF and nonlinear probability estimators. GMF has more parameters than the single Gaussian function.	GOFR	Used for classification
Roonizi and Ebadollah [28]	5	Gaussian Function Riding on the Polynomial Background. Complex realization.	Non-iterative approximation method	PRD < 0.4
Dubois et al. [12]	6 (GMF) For T wave (BiGaussian)	GMF and BiGaussian. Non-uniform Gaussian waves for different ECG components.	Generalized Orthogonal Forward Regression (GOFR)	QT (CORR = 0.92)
Elda et al. [32]	Three different polynomials (linear, quadratic, cubic polynomials)	Adaptive Multi-harmonic ECG Modeling and Interacting multiple model (IMM) with sequential Markov chain Monte Carlo (SMCMC) methods. Complex to realize.	Sequential Bayesian-based methods to effectively model and adaptively select parameters ECG modeling techniques using the interacting multiple model (IMM).	ECG-P Wave RMSE = 2.71$\times 10^{-2}$ for SMCMC and 2.29 $\times 10^{-2}$ for IMM
Proposed	7	Sum of two Gaussian functions. Simple to realize, adaptive to baseline wander, and does not stuck in local minima. However, the number of parameters is higher than single Gaussian and Bi-Gaussians.	Hybrid optimization techniques namely ApproxiGlo and ApproxiMul.	For ApproxiGlo CORR = 0.99016 RMSE = 0.02657 For ApproxiMul CORR = 0.9983 RMSE =0.0112

**Table 6:** Average results of different optimization methods used to fit normal ECG *.

Average Performance Parameters	Crauna’s Method [27]	Guo’s Method [52]	FAS Method [29]	ApproxiGlo Method	ApproxiMul Method
MSE	0.04314	0.05660	0.09497	0.00074	**7.56** $\times 10^{-5}$
NMSE	6.71 $\times 10^{-5}$	8.81 $\times 10^{-5}$	0.00015	1.17 $\times 10^{-6}$	**1.19** $\times 10^{-7}$
RMSE	0.20767	0.23791	0.30817	0.02726	**0.00869**
NRMSE	0.13454	0.15414	0.19965	0.01766	**0.00563**
CORR	0.76189	0.73095	0.58196	0.99082	**0.99907**
Runtime (in Sec.)	0.51107	0.00383	**0.0034**	4.51996	7.54061

* Note: The best performances are indicated in boldface numbers.

## Data Availability

Data can be shared upon reasonable request from the corresponding author.

## Appendix A

### Appendix A.1. Performance Evaluation Metrics

The purpose of this study was to develop a simplified mathematical model for generating an ECG waveform. The proposed model’s result optimized by hybrid optimization algorithm will be measured in the time domain, frequency domain, and time-frequency domain. These are briefly discussed below.

#### Appendix A.1.1. Time-Domain Metrics

If *x*(*n*) be the recorded or collected ECG signal and $x_{m}\left(n\right)$ be the ECG signal generated by the mathematical model; then Mean Square Error (MSE) is defined as Equation (A1).

(A1) $$MSE=\frac{1}{N}\sum_{n=0}^{N-1}{\left[x(n)-x_{m}(n)\right]}^{2}$$

The normalized form of MSE is:

(A2) $$NMSE=\frac{\sum_{n=0}^{N-1}{\left[x(n)-x_{m}(n)\right]}^{2}}{\sum_{n=0}^{N-1}{\left[x(n)\right]}^{2}}$$

Another measurement is Root Mean Square Error, which is:

(A3) $$RMSE=\sqrt{\frac{1}{N}\sum_{n=0}^{N-1}{\left[x(n)-x_{m}(n)\right]}^{2}}$$

The Normalized version of RMSE is:

(A4) $$NRMSE=\sqrt{\frac{\sum_{n=0}^{N-1}{\left[x(n)-x_{m}(n)\right]}^{2}}{\sum_{n=0}^{N-1}{\left[x(n)\right]}^{2}}}$$

#### Appendix A.1.2. Frequency-Domain Metrics

A transformation of the model and signal in the frequency domain through a Fourier Transform (FT) is done to evaluate the performance. As Fourier transform of a Gaussian function is also a Gaussian function, and the Fourier transform of a Gaussian function can be expressed as:

(A5) $$A_{i}e^{-b_{i}{\left(t-t_{i}\right)}^{2}}\overset{FT}{\overbrace{\Leftrightarrow }}\underset{Magnitude}{\underbrace{A_{i}\sqrt{\frac{\pi }{b_{i}}\;}e^{-\frac{{\left(\pi \;f\right)}^{2}}{b_{i}}}}}\;\underset{Phase}{\underbrace{e^{-j2\pi ft_{i}}}}$$

where,

(A6) $$b_{i}=\frac{1}{2B_{i}^{2}}i\in P,\;Q,R,S,T$$

As can be observed from Equation (A6), there is an inverse relationship width $b_{i}\;$ between time domain and frequency domain, because frequency domain Gaussian function $\hat{\;X}\left(\omega \right)$ is not shifted. Note that, here FT of a single Gaussian function is presented. The sum of two Gaussians like Equation (1) is also a sum of two Gaussians in the frequency domain as FT is a linear operator. Mathematically,

(A7) $$F\left[A_{1}f_{1}\left(x\right)+A_{2}f_{2}\left(x\right)\right]=A_{1}\;F_{1}\left(X\right)+A_{2}\;F_{2}\left(X\right)$$

where $\;F_{1}\left(X\right)$ and $F_{2}\left(X\right)$ are the FT of first and second Gaussian functions $f_{1}\left(x\right)$ and $f_{2}\left(x\right)$ and $A_{1}$ and $A_{2}$ are the amplitude of the Gaussians, respectively. We also used power spectral density (PSD).

#### Appendix A.1.3. Time-Frequency Domain Measure

A qualitative distortion measure named scalogram difference (ScD) is used to calculate the performance in the time-frequency domain. ScD is computed using continuous wavelet transform and defines the percentage of energy difference for each coefficient of the real and model signal. The CWT is chosen to calculate ScD due to its good time and frequency localization, which helps localize and visualize the time-varying spectral changes in the ECG signal. CWT does not suffer from cross-terms interference and presents a signal in the time-frequency plane more flexibly than Short-time Fourier Transform (STFT) or Spectrogram by applying a variable window [64]. This ScD is a two-dimensional matrix, i.e., time-scale matrix and therefore, it is a handy tool for evaluating a model performance in the time-scale domain. One can easily visualize and locate the magnitude of change between real and model ECG. The lower value represents the higher quality of a fitting [24]. If $CWT_{org}\;$ be the wavelet coefficient of the original or real signal at scale a and $CWT_{model}$ be the wavelet coefficient of the reconstructed signal of that scale, then ScD at scale a can be defined as:

(A8) $$ScD_{a}\left(i\right)=\frac{\left|CWT_{org}^{2}\left(i\right)-CWT_{model}^{2}\left(i\right)\right|}{\sum_{i=1}^{n_{a}}\left|CWT_{org}^{2}\left(i\right)-CWT_{model}^{2}\left(i\right)\right|}\times 100\%$$

where, $n_{a}$ is the total number of coefficients at scale a.

### Appendix A.2. Coefficient to Fit the with Models

The coefficient used for the GUI is shown in Table A1, Table A2, Table A3, and Table A4. Note that the model requires only the first seven, i.e., X(1) to X(7) parameters.

**Table A1:** Coefficient to fit the with BIOPAC Recorded ECG (normal ECG).

Coefficient	Global					Multi Start				
	P	Q	R	S	T	P	Q	R	S	T
X(1)	−35.820	17.820	0.691	40.690	−0.224	−0.313	−4.680	1.057	−0.500	0.345
X(2)	80.355	31.446	15.401	19.430	248.020	282.660	87.180	30.640	11.120	177.252
X(3)	65.943	20.005	14.110	12.140	46.880	43.672	19.990	14.110	18.060	92.944
X(4)	35.734	−7.800	1.057	−1.100	0.345	0.373	4.726	0.690	0.228	−0.223
X(5)	80.360	31.445	30.644	19.380	177.260	264.160	88.000	15.400	1.000	248.027
X(6)	65.782	19.957	14.110	12.190	92.949	50.571	20.580	14.110	5.676	46.880
X(7)	0.106	−0.070	−0.273	0.018	−0.001	0.011	−0.040	−0.270	0.017	−0.001
Size(samples)	300.000	88.000	48.000	77.000	429.000	300.000	88.000	48.000	77.000	429.000

**Table A2:** Coefficient to fit the model with atrial premature beat (A).

Coefficient	Global					Multi Start				
	P	Q	R	S	T	P	Q	R	S	T
X(1)	0.016	−2.044	0.729	−0.074	0.026	0.033	−0.074	0.729	−0.072	−0.083
X(2)	17.222	5.545	4.304	2.470	129.154	13.498	17.292	4.310	4.403	84.398
X(3)	3.542	5.132	3.610	1.587	45.606	7.044	3.311	3.610	0.521	14.599
X(4)	0.017	2.089	1.514	−0.074	−0.072	0.022	−0.022	1.512	−0.162	−0.034
X(5)	11.850	5.549	8.766	2.470	85.103	31.278	5.117	8.768	2.321	6.509
X(6)	3.428	5.284	3.126	1.587	12.684	10.343	1.442	3.124	1.244	44.711
X(7)	−0.046	−0.105	−0.343	−0.054	−0.043	−0.059	−0.043	−0.342	−0.053	−0.018
Size(samples)	44.000	20.000	13.000	14.000	153.000	44.000	20.000	13.000	14.000	153.000

**Table A3:** Coefficient to fit the model with paced beat (PB).

Coefficient	Global					Multi Start				
	P	Q	R	S	T	P	Q	R	S	T
X(1)	−25.17	−1.417	1.831	−0.031	13.230	0.419	−2.782	2.037	−0.081	0.140
X(2)	14.950	27.038	41.721	13.774	18.728	16.873	33.428	45.414	9.594	8.616
X(3)	3.134	14.110	27.310	7.210	16.199	4.196	12.516	27.310	7.210	16.170
X(4)	25.684	−1.417	−0.358	−0.031	−13.10	0.891	−2.222	0.549	−0.067	0.077
X(5)	14.910	27.038	1.000	13.774	18.762	12.133	17.133	14.904	19.700	30.850
X(6)	3.215	14.110	1.782	7.210	16.119	2.199	14.110	13.717	7.210	16.210
X(7)	−0.118	−0.354	0.203	0.046	−0.057	−0.117	0.528	−0.051	0.084	−0.065
Size(samples)	22.000	48.000	92.000	25.000	55.000	22.000	48.000	92.000	25.000	55.000

**Table A4:** Coefficient to fit the model with premature ventricular contraction.

Coefficient	Global					Multi Start				
	P	Q	R	S	T	P	Q	R	S	T
X(1)	−0.296	−0.054	1.335	−0.555	−0.147	0.160	−0.054	1.335	0.481	−0.067
X(2)	5.788	13.840	20.368	20.059	1.004	62.969	13.840	20.368	1.000	1.000
X(3)	14.440	4.518	4.852	24.310	29.709	10.323	4.518	4.852	2.114	4.760
X(4)	−0.126	−0.021	1.244	−0.507	0.161	0.329	−0.021	1.244	0.270	0.066
X(5)	22.613	19.132	14.064	60.740	15.084	41.613	19.133	14.065	82.000	29.099
X(6)	8.348	1.539	7.810	24.310	29.709	16.954	1.538	7.810	11.569	29.710
X(7)	0.037	−0.023	−0.099	0.142	−0.034	−0.252	−0.023	−0.099	−0.401	−0.038
Size(samples)	63.000	22.000	27.000	82.000	100.000	63.000	22.000	27.000	82.000	100.000
